# Predictors of Postpartum Hemorrhage and Associated Outcomes at a Midwest Academic Medical Center

**DOI:** 10.1089/whr.2023.0192

**Published:** 2024-04-26

**Authors:** Megan Mooberry, Natalie Voss, Linder Wendt, Kimberly A. Kenne, J. Brooks Jackson, Mary B. Rysavy

**Affiliations:** ^1^ Carver College of Medicine, University of Iowa, Iowa City, IA, USA.; ^2^ Institute for Clinical and Translational Science, University of Iowa, Iowa City, IA, USA.; ^3^ Department of Obstetrics and Gynecology, University of Iowa, Iowa City, IA, USA.; ^4^ Department of Pathology, University of Iowa, Iowa City, IA, USA.; ^5^ Department of Obstetrics, Gynecology and Reproductive Sciences, University of Texas Health Science Center at Houston, Houston, Texas, USA.

**Keywords:** postpartum hemorrhage, obstetrics, hypertension, pregnancy-induced

## Abstract

**Background:**

Postpartum hemorrhage (PPH) remains a significant cause of maternal morbidity and mortality around the world, with rates increasing in the United States. The objective of this study was to determine predictors of, and outcomes associated with, PPH at a Midwest academic health center.

**Methods:**

Demographic and clinical data were obtained from the electronic medical record on all consecutive delivering patients between May 1, 2020, and April 30, 2021. Associations between PPH and perinatal characteristics and outcomes were assessed using logistic regression models. A significance threshold of 0.05 was used for all comparisons.

**Results:**

Of the 2497 delivering patients during the study period, 437 (18%) experienced PPH. Chronic hypertension, gestational hypertension, and preeclampsia with and without severe features were all associated with increased odds of PPH (odds rations [ORs], respectively, 1.61 (95% CI:1.13–2.24, *p* = 0.006), 1.62 (95% CI 1.18–2.21, *p* = 0.003), 1.81 (95% CI 1.14–2.80, *p* ≤ 0.001), and 1.92 (95% CI 1.29–2.82, *p* = 0.009). There were also increased odds of PPH with type I diabetes: 2.83 (95% CI 1.45–5.30, *p* = 0.001), type II diabetes: 2.14 (95% CI 1.15–3.82, *p* = 0.012), twin delivery: 3.20 (95% CI 2.11–4.81, *p* ≤ 0.001), cesarean delivery: 5.66 (95% CI 4.53–7.09, *p* ≤ 0.001), and assisted vaginal delivery: 3.12 (95% CI1.95–4.88, *p* ≤ 0.001). Infants of mothers with PPH had high odds of NICU admission (CI = 1.34–2.07, *p* < 0.001) and hypoxic ischemic encephalopathy (CI = 1.64–7.14, *p* < 0.001).

**Conclusion:**

Our findings confirm previous literature that preexisting and pregnancy-related hypertension, diabetes mellitus, multiple gestation, cesarean delivery, and assisted vaginal delivery are important predictors of PPH. In addition, we found that neonates of mothers with PPH had more adverse outcomes. These results may help to inform clinical care as rates of PPH continue to rise in the United States.

## Introduction

Postpartum hemorrhage (PPH) remains the leading cause of maternal mortality globally and is a significant cause of maternal morbidity and mortality in the United States, accounting for 12.1% of maternal deaths.^1^ Aside from mortality, PPH is associated with secondary complications such as shock, disseminated intravascular coagulation (DIC), respiratory distress syndrome, acute renal failure, and Sheehan syndrome.^2^ Recent data suggest that PPH occurs in 3% of hospital deliveries in the United States, although rates vary substantially across patient populations.^3^ Multiple studies within the United States have found that the incidence of PPH has increased over the past 20 years, although the etiology remains unclear.^3,4^

There are numerous risk factors that have been associated with PPH, including placental disorders (retained placenta, placenta previa, vasa previa, and placental abruption), prolonged labor, and uterine overdistension.^3,5,6^ Other risk factors related to maternal health that have been implicated in PPH include advanced maternal age, body–mass index (BMI), preexisting or gestational diabetes mellitus, cesarean delivery, instrumented vaginal delivery, hypertension (HTN), extremes of parity, and multiple gestation.^3,6^ Although there is an abundance of literature highlighting risk factors associated with PPH, a significant subset of women who experience PPH are not identified as high-risk patients before delivery.^4,7^

Owing to the serious complications and mortality that can occur with PPH and the increasing rates of PPH in the United States, there is need for additional studies that allow for improved identification and treatment of PPH. Therefore, in this study, the primary objective was to assess predictors of PPH; secondarily, we aimed to examine maternal and neonatal outcomes associated with patients who experienced PPH at our large academic medical center in the Midwest United States.

## Materials and Methods

### Study design and definitions

This cross-sectional analysis utilized a preexisting database that was created to study COVID-19 infection during pregnancy. Prospective data were collected on every patient who delivered or underwent procedures for spontaneous or induced second-trimester abortion on the labor and delivery unit at the University of Iowa Hospital and clinics from May 1, 2020, to April 30, 2021. All deliveries, even if previable, were included in the study, as second trimester deliveries are at as high, or higher, risk of PPH as later deliveries. Maternal clinical data and demographic information were obtained from electronic medical records and double entered in a Research Electronic Data Capture (REDCap) database. Methods of sample testing for COVID-19 antibodies and results related to the primary study have been previously published.^8^

PPH was defined as blood loss exceeding 1000 mL at time of delivery or blood loss associated with signs of hypovolemia within 24 hours of delivery. Quantitative blood loss (QBL) at delivery was measured following the University of Iowa protocols, which involve volumetric measurement of blood in under-buttock drapes or surgical suction canisters and weighing blood-soaked sponges, Chux, other pads, towels, and sheets.^9^ Chronic HTN was defined as blood pressure ≥140 mmHg systolic and/or ≥90 mmHg diastolic documented before pregnancy or before 20 weeks of gestation or use of antihypertensive medications before pregnancy. Gestational HTN was defined as blood pressure ≥140 mmHg systolic and/or ≥90 mmHg diastolic on two separate occasions at least 4 hours apart after 20 weeks of gestation in a previously normotensive woman.^10,11^ Preeclampsia without and with severe features and eclampsia were defined using the American College of Obstetricians and Gynecologists (ACOG) guidelines.^11^ Stillbirth was defined as any fetus without a heartbeat before delivery. Live birth was defined as an infant born with a heartbeat. Neonatal death was defined as a live born infant who died within 28 days of delivery.

### Statistics

Summary statistics were reported for all data, with medians and interquartile ranges presented for continuous variables and with counts and percentages presented for categorical variables. We fit univariate regression models with PPH as our primary outcome and maternal demographic and clinical characteristics as predictors. A multivariate model predicting PPH was also constructed using a stepwise selection approach dictated by the Akaike Information Criterion (AIC). For this process, an intercept-only model was fit and then variables were iteratively added or removed until no variable’s addition or removal would reduce the AIC of the model. A statistical significance threshold of 0.05 was used for all comparisons, as corrections for multiple comparisons were not implemented owing to the exploratory nature of the analysis. All statistical analyses were conducted using R, version 4.3.1.

### Ethical considerations

This study was approved by the Institutional Review Board at the University of Iowa (IRB ID#: 202004278).

## Results

A total of 2497 patients delivered 2604 infants at the University of Iowa Hospitals and Clinics during the study period. Population characteristics, complications, and delivery information were previously published,^12^ but are included here for easier reference (Table 1). A total of 437 women (18%) experienced PPH: 103 (4.1%) had quantitative blood loss (QBL) more than 1500 mL, 34 (1.4%) had QBL more than 2000 mL, and 17 (0.7%) had QBL more than 2500 mL.

**Table 1:** Population Characteristics, Complications, and Delivery Information^12^

Characteristic, complication, delivery information	*N* (%), except as noted
Total number of patients	2497
Quantitative blood loss at delivery (mL) mean, IQR)	325 (IQR 150, 687)
Postpartum hemorrhage (QBL > 1000 mL)	437 (17.5%)
Maternal age (mean, IQR)	30 (IQR 26, 33)
Race (self-reported)	
White	1766 (70.7%)
African American/black	305 (12.2%)
Black African	2 (<0.1%)
Hispanic/Latino	211 (8.5%)
American Indian/Alaska Native	6 (0.2%)
Native Hawaiian/Pacific Islander	5 (0.2%)
Asian	116 (4.6%)
Multiracial (two or more races)	54 (2.2%)
Unknown/unspecified	1 (<0.1%)
Decline to answer	31 (1.2%)
Patient-preferred language	
English	2273 (91.0%)
Spanish	77 (3.1%)
French	59 (2.4%)
Arabic	27 (1.1%)
Other	61 (2.4%)
Insurance	
Private (only)	1286 (51.5%)
Medicaid/Medicare (any)	1, 140 (45.7%)
Other	3 (0.1%)
None	68 (2.7%)
Prenatal care	
No	13 (0.5%)
Yes	2482 (99.4%)
Unknown	2 (<0.1%)
Gravidity (upon admission) (mean, IQR)	2 (IQR 1, 18)
Parity (upon admission) (mean, IQR)	1 (IQR 0, 11)
Diabetes	
No	2113 (84.6%)
Type I	42 (1.7%)
Type II	54 (2.2%)
Gestational	288 (11.5%)
Hypertensive disorder during pregnancy or delivery	
No	1912 (76.6%)
Chronic hypertension	181 (7.2%)
Gestational hypertension	196 (7.8%)
Preeclampsia without severe features	104 (4.2%)
Preeclampsia with severe features	162 (6.5%)
Eclampsia	1 (<0.1%)
BMI at admission (mean, IQR)	32 (IQR 28, 37)
Number of fetuses	
Singleton	2393 (95.8%)
Twins	102 (4.1%)
Triplets	2 (<0.1%)
Gestational age at delivery (mean, IQR)	39w0d (IQR 37w3d, 39wk5d)
Preterm birth	466 (18.7%)
Preterm labor	325 (13.0%)
Premature rupture of membranes>	246 (9.9%)
Morbidly adherent placenta (accreta, percreta, increta)	3 (0.1%)
Placental abruption	41 (1.6%)
Chorioamnionitis	167 (6.7%)
Nonreassuring fetal status	289 (11.6%)

IQR, interquartile range; QBL, quantitative blood loss; BMI, body–mass index.

Maternal and neonatal characteristics and their associations with PPH are listed (Table 2). Patients with preexisting diabetes were more likely to experience hemorrhage relative to patients with no diabetes; type I diabetics had 2.83 higher odds of PPH (CI = 1.45–5.30, *p* = 0.001) and type II diabetics had 2.14 higher odds of PPH (CI = 1.15–3.82, *p* = 0.012). However, gestational diabetes did not have increased odds of PPH. A diagnosis of HTN, including chronic HTN, gestational HTN, and preeclampsia, significantly increased a patient’s odds of experiencing PPH, whereas not having HTN was associated with decreased odds of PPH. Patients with a parity of 1–4 had lower odds of PPH (OR = 0.73, CI = 0.59–0.90, *p* = 0.04) compared with nulliparous patients. Increasing maternal age was associated with higher odds of PPH (OR = 1.12, CI = 1.01–1.23, *p* = 0.024), and maternal age >40 years was associated with 2.25 higher odds of PPH (CI = 1.18–4.12, *p* = 0.011) compared with younger patients.

**Table 2.** Predictors of Postpartum Hemorrhage: Univariate Analysis

Characteristic	N	N (%)	Number of PPH among subgroups (%)	OR	95% CI	*p*-value
Diabetes	2497					
Type 1 diabetes		42 (1.7%)	15 (36%)	2.83	1.45, 5.30	**0.001**
Type 2 diabetes		54 (2.2%)	16 (30%)	2.14	1.15, 3.82	**0.012**
Gestational diabetes		288 (12%)	59 (20%)	1.31	0.96, 1.77	0.085
Hypertension (HTN)	2497					
No HTN		1922(77%)	295 (15%)	0.55	0.44, 0.69	**<0.001**
Chronic HTN		199 (8.0%)	49 (25%)	1.61	1.13, 2.24	**0.006**
Gestational HTN		240 (9.6%)	59 (25%)	1.62	1.18, 2.21	**0.003**
Preeclampsia without severe features		103 (4.1%)	28 (27%)	1.81	1.14, 2.80	**0.009**
Preeclampsia with severe features		135 (5.4%)	38 (28%)	1.93	1.29, 2.82	**<0.001**
Eclampsia		1 (<0.1%)	0 (0%)	0.00		>0.9
Insurance status	2497					
Private (only)		1286(52%)	242 (19%)	—	—	
Medicaid/Medicare		1140(46%)	179 (16%)	0.80	0.65, 0.99	**0.043**
Other		3 (0.1%)	0 (0%)	0.00		>0.9
Primary language spoken						
English	2497	2273 (91%)	386 (17%)	0.69	0.5, 0.97	**0.030**
Race						
White	2397	1766 (71%)	293 (17%)	0.81	0.65, 1.01	0.063
Maternal age (5-year increase)	2497	—	—	1.12	1.01, 1.23	**0.024**
Maternal age >35	2497	460 (18%)	94 (20%)	1.27	0.98, 1.63	0.067
Maternal age >40	2497	47 (1.9%)	15 (32%)	2.25	1.18, 4.12	**0.011**
Maternal BMI (5-unit increase)	2492	—	—	1.16	1.10, 1.23	**<0.001**
Parity						
0		951 (38%)	195 (21%)	—	—	
1–4		1493 (60%)	237 (16%)	0.73	0.59, 0.90	**0.004**
≥5		53 (2.1%)	5 (9.4%)	0.40	0.14, 0.94	0.057
Premature rupture of membranes	2497	246 (9.9%)	42 (17%)	0.97	0.67, 1.36	0.9
Gestational age	2496					
< 20 weeks		16 (0.6%)	2 (13%)	—	—	
20–27 weeks		81 (3.2%)	8 (9.9%)	0.77	0.17, 5.44	0.8
28–31 weeks		50 (2.0%)	8 (16%)	1.33	0.29, 9.53	0.7
32–36 weeks		319 (13%)	77 (24%)	2.23	0.60, 14.4	0.3
>36 weeks		2030 (81%)	342 (17%)	1.42	0.39, 9.06	0.6
Plurality	2497					
1		2392 (95.8%)	396 (17%)	—	—	
2		103 (4.1%)	40 (39%)	3.20	2.11, 4.81	**<0.001**
3		2 (<0.1%)	1 (50%)	5.04	0.20, 128	0.3
Live birth	2604	2553 (98.0%)	474 (19%)	2.08	0.91, 1.64	0.12
Stillbirth	2604	32 (1.2%)	4 (13%)	0.52	0.12, 1.48	0.3
Mode of delivery	2604					
Vaginal		1554 (60%)	134 (8.6%)	—	—	
Assisted-vaginal (vacuum or forceps)		123 (4.7%)	28 (23%)	3.12	1.95, 4.88	**<0.001**
Cesarean		908 (35%)	316 (35%)	5.66	4.53, 7.09	**<0.001**
D&C/ D&E		19 (0.7%)	1 (5.3%)	0.59	0.03, 2.88	0.6
Birth weight	2579	—	—	1.00	1.00, 1.00	0.081

Statistically significant *p*-values are bolded.

OR, odds ratio; CI, confidence interval; HTN, hypertension; D&C/D&E, dilation and curettage/dilation and evacuation.

Statistically significant associations were found between PPH and mode of delivery, with cesarean deliveries having more than fivefold higher odds of PPH (OR = 5.66, CI = 4.53–7.09, *p* ≤ 0.001) and assisted vaginal delivery having more than threefold higher odds of PPH (OR = 3.12, CI = 1.95–4.88, *p* ≤ 0.001) than those patients who had spontaneous vaginal deliveries.

Maternal age >35 years, preterm delivery, and premature rupture of membranes were not significantly associated with increased odds of PPH.

Maternal characteristics analyzed in a multivariate analysis are included (Table 3). This model includes maternal age, parity (categorized), plurality (number of babies delivered), primary language, gestational HTN, preterm labor, and unscheduled cesarean section as predictors. Unscheduled cesarean delivery (OR = 4.51, CI = 3.56–5.70, *p* < 0.001), plurality (OR = 3.45, CI = 2.21–5.38, *p* < 0.001), gestational HTN (OR = 1.52, CI = 1.08–2.12, *p* = 0.014), and increased maternal age (OR = 1.19, CI = 1.07–1.32, *p* = 0.002) were all significantly associated with increased odds of PPH in the multivariable model. Preterm labor was significantly associated with decreased odds of PPH (OR = 0.68, CI = 0.48–0.95, *p* = 0.026). Parity was not found to be significantly associated with PPH in the multivariate analysis.

**Table 3.** Predictors of Postpartum Hemorrhage: Multivariate Analysis

Characteristic	OR	95% CI	*p*-value
Maternal age (5-year increase)	1.19	1.07, 1.32	**0.002**
Parity			
0	—	—	
1–4	0.82	0.65, 1.04	0.10
≥5	0.46	0.15, 1.12	0.12
Plurality	3.45	2.21, 5.38	**<0.001**
Language			
Other	—	—	—
English	0.67	0.47, 0.96	**0.025**
Race			
Other	—	—	—
White	0.81	0.62, 1.05	0.11
Diabetes			
No	—	—	—
Type 1 diabetes	2.79	1.41, 5.32	**0.002**
Type 2 diabetes	1.84	0.97, 3.36	0.053
Gestational diabetes	1.20	0.87, 1.65	0.3
No hypertension (antepartum/intrapartum)	0.62	0.49, 0.79	**<0.001**
Unscheduled cesarean delivery	4.51	3.56, 5.70	**<0.001**

Statistically significant *p*-values are bolded.

OR, odds ratio; CI, confidence interval.

Neonatal outcomes associated with PPH included NICU admission, diagnosis of hypoxic–ischemic encephalopathy, and use of therapeutic hypothermia (Table 4).

**Table 4.** Neonatal Outcomes Associated with Postpartum Hemorrhage (*n* = 2604)

Outcome	N (%)	OR	95% CI	*p*-value
NICU admission	638 (25%)	1.67	1.34, 2.07	**<0.001**
Sepsis	131 (5.0%)	1.16	0.74, 1.77	0.5
Hypoglycemia	605 (23%)	1.19	0.95, 1.50	0.13
Diagnosis of hypoxic ischemic encephalopathy	30 (1.2%)	3.46	1.64, 7.14	**<0.001**
Therapeutic hypothermia	23 (0.9%)	2.89	1.20, 6.62	**0.014**
Neonatal death (< 28 days of life)	41 (1.6%)	0.61	0.21, 1.43	0.3

Statistically significant *p*-values are bolded.

OR, odds ratio; CI, confidence interval; DVT, deep vein thrombosis; PE, pulmonary embolism.

## Discussion

The rate of PPH in patients delivering at University of Iowa Hospitals & Clinics was 18%, which was well above the national average of approximately 3%.^4^ This higher rate likely reflects our high-risk patient population, as University of Iowa Hospitals & Clinics is a regional referral center accepting the transfer of high-risk patients from around the state of Iowa and neighboring states. Although there are not a large number of studies reporting PPH rates for a single academic or tertiary hospital with a similar high-risk population, our rate was almost exactly the same as a study from a similar time period during the early COVID-19 pandemic at an academic center in Italy (total PPH rate of 18.3%),^13^ and just slightly higher than some other reported results from tertiary care centers in the United States which reported PPH rates of 10.3 and 12.3%.^14,15^

Uterine atony is widely recognized as the leading cause of PPH. One factor contributing to uterine atony is uterine overdistension, which can be caused by multiple gestation, increased maternal parity, and/or fetal macrosomia.^5^ A total of 103 sets of twins were delivered during our study period, comprising 4% of all deliveries. Patients had 3.20 times higher odds of PPH when delivering twins, compared with singleton deliveries. In our univariate analysis, this relationship was not statistically significant in triplets, but we had only 2 patients deliver triplets during our study period. In our multivariate analysis, each additional infant delivered by a mother more than tripled the odds of PPH. This finding is similar to other studies and is likely explained by uterine overdistension and the upward of 20% increase in cardiac output that occurs as a result of multiple gestation when compared with singleton gestation.^16,17^

Moreover, 38% of the delivering women in our cohort were nulliparous, whereas 62% were multiparous. Results from our study show that nulliparous women had a higher risk of PPH. This finding is consistent with other studies and is likely owing to differences in labor in nulliparous women.^18,19^ In the multivariate analysis, parity was not significantly associated with PPH. Previous studies have suggested that grand multiparity, or parity >5, is a risk factor for PPH.^20^ This relationship was not found in our study in univariate or multivariate analysis, although this group represented only 2% of our total cohort. The reason for these findings is unclear; it may be due to the small number of grand-multiparous patients, or it may represent differences in identification and management, if patients are identified as high risk and treated differently. We did not collect data on patterns of PPH management.

Many studies have noted that preexisting maternal health conditions, such as preexisting diabetes mellitus and HTN, are associated with increased rates of PPH. In our cohort, 1.7% of delivering patients had type 1 diabetes and 2.2% had type 2 diabetes. Similar to previous studies, we found an increased rate of PPH in women with pregestational diabetes mellitus compared with women without diabetes mellitus. This increased rate may, in part, be due to the increased incidence of fetal macrosomia in patients with pregestational diabetes.^21,22^ Macrosomia increases the risk for uterine overdistension and thus atony, cesarean section, fourth-degree perinatal lacerations, and chorioamnionitis, all of which have been associated with PPH.^21,23^ The birth of macrosomic infants has in other studies been shown to increase the odds of postpartum bleeding and genital tract injury by 3–5 times.^21,24^ In our study, for every 500 g increase in birth weight, there were 1.06 increased odds of PPH; however, this did not reach statistical significance. In previous studies, gestational diabetes has been indicated as a risk factor for PPH owing to the same mechanism of macrosomia.^21,24^ Interestingly, our study did not find gestational diabetes to be a significant predictor of PPH. It is worth noting that preexisting diabetes mellitus has also been indicated as an independent predictor of PPH, and while the exact mechanism is unclear, it has been hypothesized that diabetes may alter vascular integrity and directly contribute to the development of atony and/or hemorrhage.^6^

About 1 out of every 4 women in our study had some form of HTN; 8.0% had chronic HTN, 9.6% had gestational HTN, 4.1% had preeclampsia without severe features, 5.45% had preeclampsia with severe features, and <0.1% patient had eclampsia. Some of these patients progressed from chronic or gestational HTN to more severe forms of hypertensive disorders of pregnancy. The large number of hypertensive patients likely reflects referral patterns to the hospital, as many patients may be transferred in labor owing to hypertensive diseases at early gestational ages, or if there is a need for complex care unavailable at smaller hospitals. As expected, all forms of HTN showed statistically significant associations with increasing odds of PPH, except for eclampsia, of which we only had 1 case. Increased mean arterial pressure, as found in hypertensive disorders, has previously been associated with greater blood loss after delivery.^25^ In addition, it has been proposed that hypertensive disorders during pregnancy may cause abnormal placentation and alter vascular physiology, leading to increased risk of PPH.^6,26^ However, we only had 3 cases of morbidly adherent placenta during the study period, and so, it was not possible to draw any significant conclusions on this topic. Finally, the administration of magnesium sulfate to prevent eclampsia may contribute to increased rates of PPH in patients being treated for preeclampsia with severe features, as some studies have found magnesium sulfate to be associated with uterine atony and subsequent PPH.^27^ Importantly, results from our study found that mothers who had no history of HTN had significantly lower odds of experiencing PPH.

In analyzing the mode of delivery, we found that cesarean delivery and assisted-vaginal delivery were associated with higher odds of PPH. These findings are consistent with other studies.^5,28,29^ Cesarean delivery is a major abdominal surgery accompanied by an intraoperative risk of bleeding.^29^ In addition, unscheduled cesarean sections may be necessary owing to placental abnormalities, blood disorders, or preeclampsia, all of which carry a separate risk of PPH.^29^ In our analysis, unscheduled cesarean delivery was associated with increased odds of PPH compared with planned cesarean delivery. Assisted-vaginal delivery may cause genital tract trauma, which may increase bleeding following delivery.^5^

Other maternal characteristics that have been associated with PPH include BMI and age. In a previous article that included part of this same population, we found that increasing maternal BMI at delivery was associated with increased rates of PPH in our cohort.^30^ This was particularly true in patients with a BMI over 40 who had 1.7 times higher odds of experiencing PPH compared with normal-weight controls.^30^ Our present analysis, also using maternal BMI at delivery, found that every BMI increase of 5 units was associated with increased odds of PPH. It was previously believed that this association could be explained by increased rates of cesarean delivery and comorbidities among obese patients.^31^ However, other studies controlling for these factors have found high maternal BMI to be an independent risk factor for PPH, likely attributable to increased uterine atony among these patients,^32,33^ although why these women are more at risk of atony remains unclear. We only had information on BMI at the time of delivery, owing to the large number of patients transferred to us for delivery care after receiving prenatal care elsewhere. Prepregnancy BMI, as well as total weight gain in pregnancy and the association of these factors with PPH, is an excellent area for further study.

Previous literature analyzing maternal age and its association with PPH has yielded mixed results. Some studies have found advanced maternal age, defined as a pregnancy occurring in a woman >35 years, to be associated with an increased incidence of PPH.^34–36^ This finding has been attributed to decreased vascular integrity, myometrial atrophy, and increased medical comorbidities.^37,38^ Other studies have reported that advanced maternal age has a protective effect on PPH when controlling for other risk factors such as HTN, diabetes mellitus, and cesarean delivery.^39^ Our analysis showed a statistically significant relationship between maternal age as a continuous variable and PPH. In further analyzing advanced maternal age, 18% of our cohort was >35 years, but we did not find a significant association between PPH and this age group, suggesting that 35 may not represent a clear cutoff point for increased risk of PPH. However, we did find that maternal age >40 years was significantly associated with increased odds of PPH. In addition, in the multivariate analysis, increased age as a continuous variable was found to be a statistically significant predictor of PPH.

The relationship between gestational age and PPH remains unclear. Some studies describe an increased risk in PPH following post-term deliveries, a phenomenon likely explained by increased infant size and/or reduced myometrial contractility contributing to uterine atony.^40–42^ Other studies have found increased rates of PPH in preterm deliveries owing to increased risk of retained placenta, a known predictor of PPH.^43^ Still, other studies have found no relationship between gestational age and PPH.^44^ We did not find a significant association between gestational age and PPH.

Premature rupture of membranes (PROM) has been found to be a risk factor for PPH.^45^ This finding is likely explained by the increased rates of cesarean section and intraamniotic infection associated with PROM, both of which have been associated with PPH.^46,47^ Despite the fact that 10% of all patients in the study experienced PROM, we did not find a statistically significant relationship between PPH and this cohort. In our population, we did not specifically separate out preterm PROM from term PROM, which may have contributed to our findings. Further investigation regarding this relationship is needed.

Historically, women who identify as a racial or ethnic minority have experienced the greatest burden of severe maternal morbidity, including increased rates of hemorrhage, stroke, embolism, and other serious complications.^48^ Previous literature has found Hispanic ethnicity and Asian race to be associated with higher rates of atonic PPH.^6^ Our study did not find race to be a significant predictor of PPH, although we may have been limited by our relatively small sample size of patients identifying as racial or ethnic minorities, as 70.7% of our study population self-identified as White. In addition, a previous study found that women who speak a primary language besides English were less likely to receive prenatal care and more likely to have severe maternal morbidities.^49^ Our study corroborates these findings, as women who utilized English as their primary language were found to have lower rates of PPH. When analyzing insurance status, 52% of our study population had private insurance and 46% had public insurance (Medicaid/Medicare). We found that women with Medicaid or Medicare had lower odds of experiencing PPH compared with women with private insurance. This finding differs from a previous study, which found that women with private insurance have lower rates of severe morbidity.^50^ The reason for our findings remains unclear; public insurance is typically used as a marker for lower socioeconomic status, which has previously been linked to higher rates of maternal morbidity and mortality.^50,51^

Stillbirth has also been reported as a predictor of PPH.^52,53^ However the exact etiology remains unknown. There may be underlying conditions that are implicated in both stillbirth and PPH, such as preeclampsia or obstructed labor without proper care.^52,54^ Stillbirth has also been associated with retained placenta and DIC, which are both known risk factors of PPH.^43,55,56^ We did not find a significant association between stillbirth and PPH, however, this is likely due to the low number of stillbirths in our cohort.

In addition to maternal predictors of PPH, we also examined neonatal outcomes associated with PPH. We found that infants born to mothers experiencing PPH were more likely to have hypoxic– ischemic encephalopathy, require therapeutic hypothermia, and require NICU admission. We believe that these findings may be related to the reasons the maternal patient experienced PPH, such as hypertensive diseases, diabetes, or mode of delivery; however, we found no other literature on this topic, and so, this highlights gaps in the existing literature, which primarily focuses on maternal outcomes following PPH.

The strengths of this study include having data collected from all consecutive deliveries over a 1-year period and the use of double data entry to ensure accuracy. An additional strength was our use of an easily definable outcome, PPH, which was measured using a consistent clinical protocol. We were limited by the single-site nature of this study, which may affect the generalizability of our findings.

## Conclusion

In a cohort of 1 year of consecutive deliveries at a large academic referral hospital in the Midwest, we found that preexisting and pregnancy-related HTN, preexisting diabetes mellitus, multiple gestation, cesarean delivery, and assisted vaginal delivery were important predictors of PPH. Although our findings largely serve to confirm the results of other studies, they also help to inform clinical care for the future with more recent patient data, as PPH remains a significant contributor to maternal morbidity and mortality in the United States. Knowing predictors of PPH may help clinicians to identify patients most at risk of PPH before delivery and thus be more prepared to quickly identify and treat hemorrhage.

## Abbreviations Used

ACOG: American College of Obstetricians and Gynecologists
AIC: Akaike Information Criterion
BMI: body mass index
CI: confidence interval
D&C: dilation and curettage
D&E: dilation and evacuation
DIC: disseminated intravascular coagulation
HTN: hypertension
NICU: neonatal intensive care unit
OR: odds ratio
PPH: post-partum hemorrhage
PROM: premature rupture of membranes
QBL: quantitative blood loss
